# Hierarchical NiV-LDH Nanosheet Arrays Vertically Grown on MXene-Embedded Carbon Nanofibers for High-Performance Flexible Supercapacitors

**DOI:** 10.3390/nano16161014

**Published:** 2026-08-17

**Authors:** Deyang Zhang, Wenbo Guo, Binhe Feng, Yikai Ge, Tao Peng, Jinbing Cheng, Paul K. Chu

**Affiliations:** 1 Henan Joint International Research Laboratory of New Energy Storage Technology, Xinyang Normal University, Xinyang 464000, China; 17550091825@163.com (W.G.); fengbinhe2012@163.com (B.F.); 19836973063@163.com (Y.G.); tpeng@xynu.edu.cn (T.P.); 2 Henan International Joint Laboratory of MXene Materials Microstructure, Collaborative Innovation Center of Intelligent Explosion-Proof Equipment of Henan Province, Nanyang Normal University, Nanyang 473061, China; chengjinbing1988@163.com; 3 Department of Physics, Materials Science & Engineering, and Biomedical Engineering, City University of Hong Kong, Tat Chee Avenue, Kowloon, Hong Kong, China; paul.chu@cityu.edu.hk

**Keywords:** NiV-LDH, MXene, electrospinning, supercapacitors, flexible electrodes

## Abstract

A flexible integrated composite electrode is fabricated using NiV-layered double hydroxide (NiV-LDH) nanosheets grown perpendicularly onto a Ti_3_C_2_T_x_ MXene-incorporated carbon nanofiber scaffold (MXene/CNFs). This hybrid structure, prepared by electrospinning and a hydrothermal treatment, is referred to as NiV-LDH@MXene/CNFs. Constructed from a conductive MXene/CNF scaffold and vertically aligned NiV-LDH nanosheets, the integrated flexible electrode offers uninterrupted electron transport, good flexibility, abundant active sites, and strong interfacial cohesion, thereby obviating the use of polymeric binders and conductive additives. The hydrophilic nature of MXene and the three-dimensionally interconnected porous structure favor rapid electrolyte uptake and ion diffusion. As a result of these synergistic effects, the composite exhibits a specific capacitance of 614 F g^−1^ at 1 A g^−1^ and retains 60% of its initial capacitance after 10,000 cycles at 5 A g^−1^ in a three-electrode cell. An asymmetric supercapacitor made of this material and activated carbon achieves 68.75% capacitance retention under the same cycling protocol at 5 A g^−1^ and shows a stable open-circuit voltage of 1.37 V. Two cells in series are capable of lighting a 3 V LED strip. Overall, this work validates an effective strategy to prepare high-capacity, robust, and binder-free flexible electrodes for advanced energy-storage applications.

## 1. Introduction

Increasingly rigorous demands are being placed on energy storage systems by the rapid spread of wearable and portable electronics, which require flexibility, light weight, and a high energy density [1]. Owing to their high power density, long cycle life, and fast charge/discharge capability, flexible supercapacitors have drawn considerable attention in energy storage technologies [2]. The electrochemical properties of supercapacitors are largely determined by the electrode materials. In this regard, nickel–vanadium layered double hydroxide (NiV-LDH) is recognized as a promising pseudocapacitive material due to the abundant electroactive sites, high theoretical specific capacitance, and layered structure that enables ion intercalation/deintercalation [3]. However, the practical application of NiV-LDH is hampered by its poor intrinsic electronic conductivity and substantial volumetric expansion during repeated charging/discharging cycles, resulting in structural degradation and rapid capacity loss [4]. To overcome these drawbacks, extensive efforts have been made to combine LDH materials with highly conductive MXene nanosheets [5]. For example, Li et al. have synthesized MXene/NiCu-LDH composite thin films by electrostatic self-assembly and shown a specific capacitance of 556.4 F g^−1^ at 1 A g^−1^ [5]. Another study has reported a MXene@NiCo-LDH composite with a specific capacitance of 586.2 F g^−1^ at 1 A g^−1^ [6]. These studies demonstrate that the incorporation of MXene can inhibit the agglomeration of LDH nanosheets, enhance electrical conductivity, and offer more available active sites [7,8]. In particular, the integration of MXene with carbon nanofibers (CNFs) by electrospinning is a versatile strategy to construct flexible, self-standing electrodes [9]. For instance, electrospinning of MXene with (polyacrylonitrile) PAN followed by carbonization can form a three-dimensional conductive network of MXene/CNFs that integrates the high electrical conductivity of MXene with the flexible nature of a self-supporting carbon framework [10]. Wang et al. have demonstrated that electrodeposition of MnO_2_ on MXene/CNFs films improves the capacitive performance [11]. However, despite these achievements, the ordered and durable growth of NiV-LDH nanostructures on MXene/CNF networks and the adequate realization of the mutual effects between the two components to both enhance specific capacitance and cycling stability remain a challenge that requires further investigation [11].

Layered double hydroxides (LDHs) consist of positively charged brucite-like layers with interlayer anions and water molecules, and their unique 2D structure enables reversible intercalation/deintercalation of electrolyte ions, contributing to the pseudocapacitive charge storage. The tunable metal composition in the hydroxide layers allows for versatile redox chemistry, making LDHs attractive electrode materials for supercapacitors. However, the practical application of LDHs is severely hindered by their poor intrinsic electronic conductivity and the tendency for nanosheet agglomeration, which limit the utilization of active sites and lead to unsatisfactory rate capability and cycling stability. To address these challenges, extensive efforts have been devoted to integrating LDHs with conductive carbonaceous substrates. For instance, Roy et al. electrochemically deposited NiMn-LDH onto exfoliated graphite, achieving significantly enhanced energy and power densities in a battery–supercapacitor hybrid system [12]. In another study, CoMn-LDH was formed within electrochemically exfoliated graphite via a single-step process, demonstrating that the intimate contact between LDH and the conductive carbon scaffold effectively facilitates electron transfer and improves the overall electrochemical performance [13]. These studies highlight the critical role of conductive substrates in unlocking the full potential of LDH materials.

Inspired by the above considerations, the construction of a NiV-LDH@MXene/CNFs composite electrode featuring a three-dimensional network architecture, by in situ hydrothermal growth of NiV-LDH nanosheets on the MXene/CNFs film. This design rationally combines the respective advantages of each component. The MXene/CNFs scaffold offers continuous electron transport paths and mechanical flexibility, whereas the in situ grown NiV-LDH nanoarrays shorten the ion diffusion distances and expose abundant electrochemically active sites, thus maximizing the capacitive contribution of NiV-LDH. Consequently, the composite electrode displays high specific capacitance, superior rate capability, and favorable cycling stability during electrochemical measurements. To demonstrate the practicality, an asymmetric supercapacitor cell assembled with NiV-LDH@MXene/CNFs as the cathode and activated carbon as the anode is shown to deliver commendable energy storage performance and practical applicability. This study presents a feasible route to constructing high-performance flexible supercapacitor electrodes by the synergistic integration of MXene/CNF conductive frameworks and LDH active materials.

## 2. Results and Discussion

### 2.1. Synthesis and Characterization

**Figure 1:**
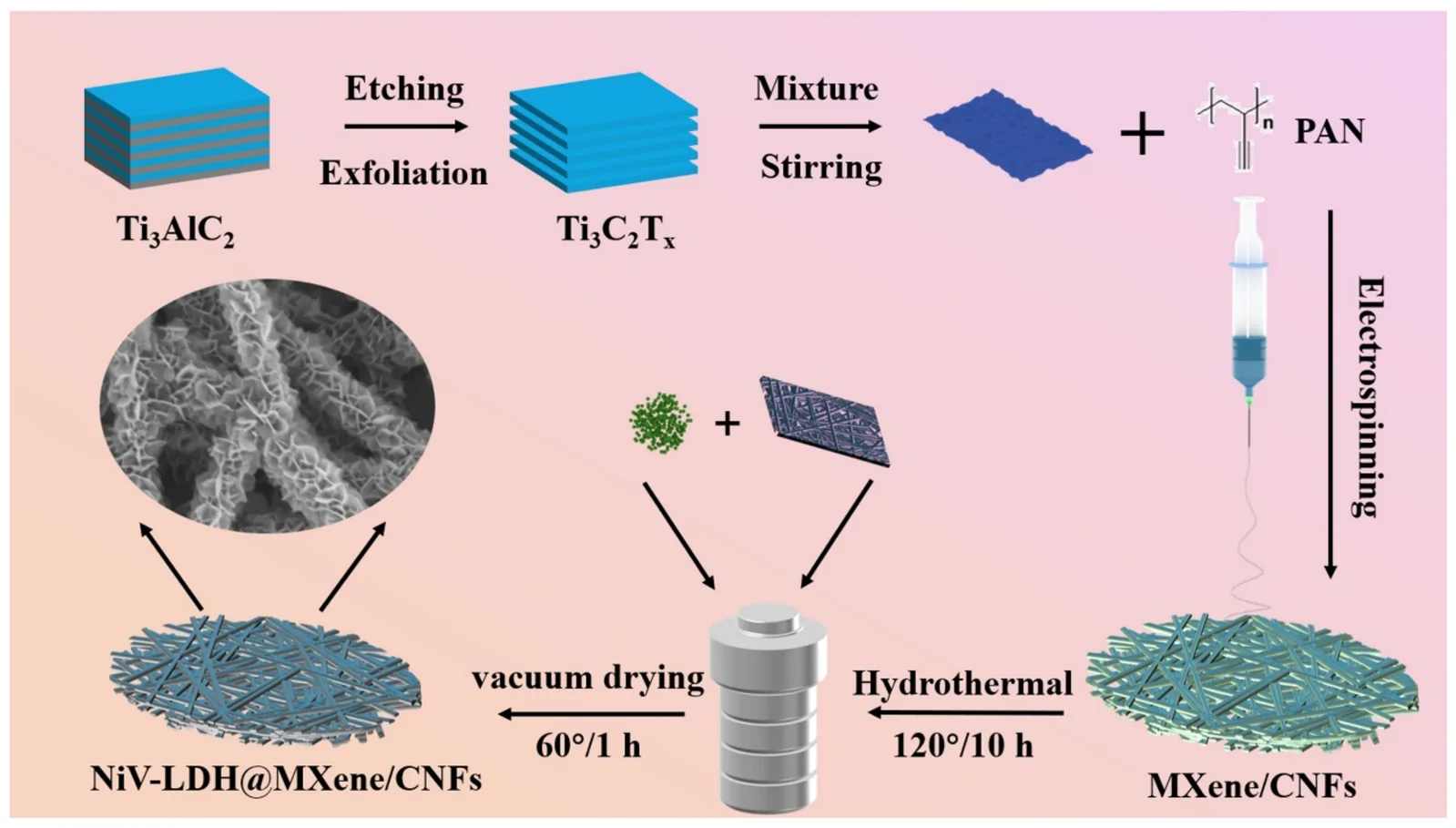
Diagram of the preparation of NiV-LDH@MXene/CNFs carbon nanofibers.

The preparation of the NiV-LDH@MXene/CNFs composite is shown schematically in Figure 1. The Ti_3_AlC_2_ MAX phase ceramic powder is the initial material, from which the Al layers are selectively removed by etching with a LiF/HCl mixed solution, followed by ultrasonic exfoliation to produce few-layered Ti_3_C_2_T_x_ MXene nanosheet dispersions. The obtained MXene was then blended with polyacrylonitrile (PAN) to prepare the spinning precursor solution, which was subsequently processed into MXene/PAN composite fiber films by electrospinning. After pre-oxidation and carbonization, the films were converted into flexible, self-supporting MXene/CNFs substrates featuring a three-dimensional conductive network (Figure S2) [14]. Thereafter, the pre-cut MXene/CNFs films were immersed in a hydrothermal reaction system containing a nickel source, vanadium source, urea, and citric acid, and maintained at 120 °C for 10 h to allow in situ growth of NiV-LDH nanosheets on the carbon fibers [15]. After the reaction, the composite films were washed and dried to form the flexible NiV-LDH@MXene/CNFs electrode.

**Figure 2:**
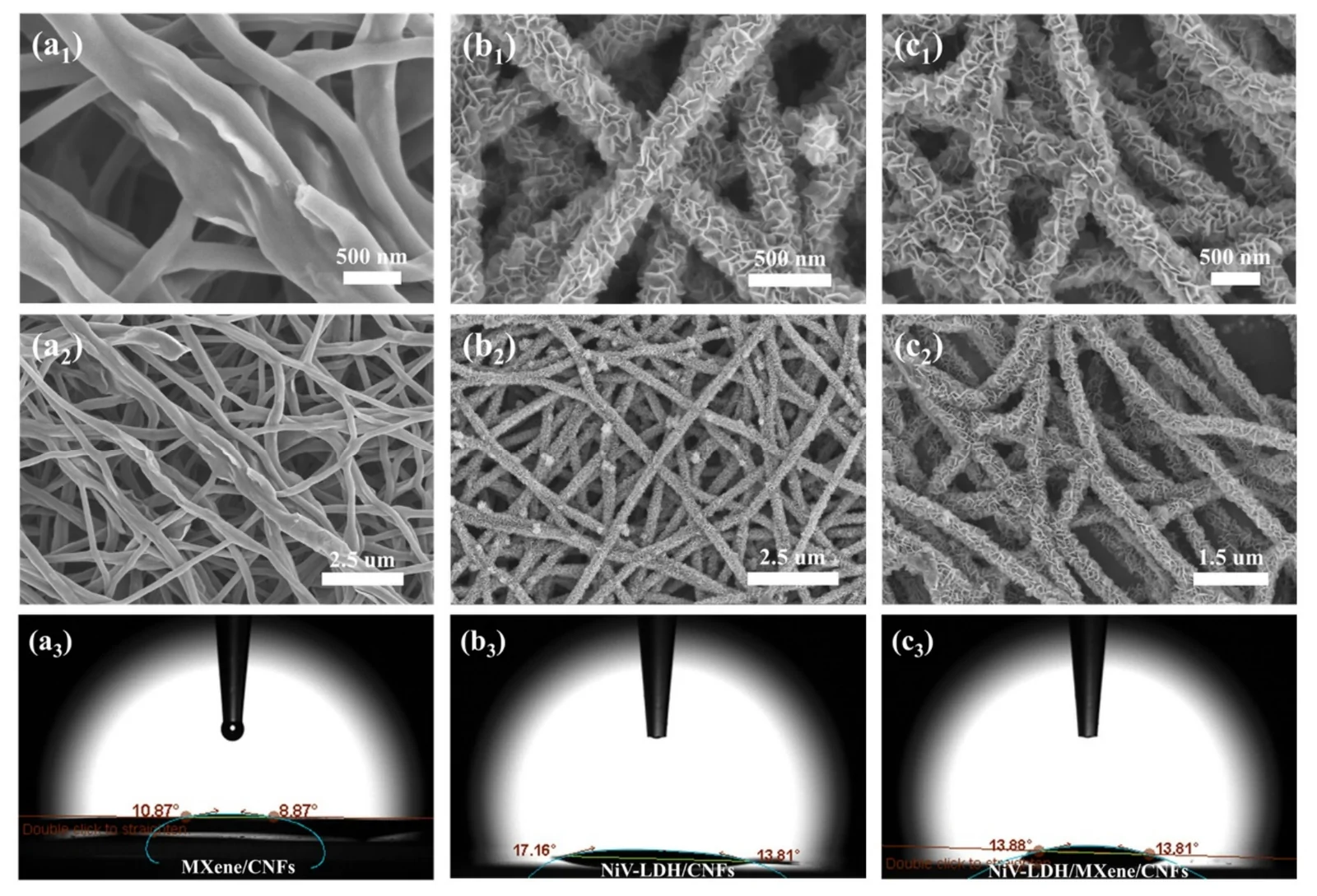
(**a1,a2**) SEM images of MXene/CNFs; (**b1**,**b2**) SEM images of NiV-LDH/CNFs; (**c1**,**c2**) SEM images of NiV-LDH@MXene/CNFs; (**a3**–**c3**) images showing the surface contact angles on MXene/CNFs, NiV-LDH/CNFs, and NiV-LDH@MXene/CNFs.

Figure 2 presents the scanning electron microscopy (SEM) morphology of the three electrode materials, together with contact angle measurements to evaluate the surface hydrophilicity. Figure 2(a1,a2) displays the morphology of the MXene/CNFs flexible substrate. After electrospinning and carbonization, Ti_3_C_2_T_x_ MXene nanosheets are uniformly coated on the carbon fiber surfaces to form a continuous three-dimensional conductive network. The MXene nanosheets adhere tightly to the fibers without obvious agglomeration. Figure 2(b1,b2) depicts the SEM images of the NiV-LDH/CNFs sample obtained by hydrothermally growing NiV-LDH on traditional carbon fibers (PAN/CNFs). The NiV-LDH nanosheets are anchored on the carbon fiber surfaces.

Figure 2(c1,c2) presents the SEM images of the NiV-LDH@MXene/CNFs composite electrode. Similar to the NiV-LDH/CNFs sample, NiV-LDH nanosheets are grown on the carbon fiber surfaces to form nanosheet arrays. From a morphological perspective, no essential difference is observed between the two samples, both exhibiting the characteristic lamellar morphology of NiV-LDH. However, Figure 2(a3–c3) shows that the NiV-LDH@MXene/CNFs composite has a contact angle of 13.845°, which is much smaller than the 15.485° on NiV-LDH/CNFs. This difference originates mainly from the rich surface functional groups of MXene, which endow the composite with hydrophilic properties. In addition, this hydrophilic feature remains largely intact after subsequent NiV-LDH deposition [16]. Additionally, the negatively charged surface groups of MXene provide ample nucleation centers for LDH formation, while the heterointerface established between them favors charge transfer [17]. In the meantime, the superior electrical conductivity of MXene offsets the poor inherent conductivity of LDH [18]. These effects are expected to substantially enhance the electrochemical performance of the composite electrode, translating into superior specific capacitance and rate capability.

**Figure 3:**
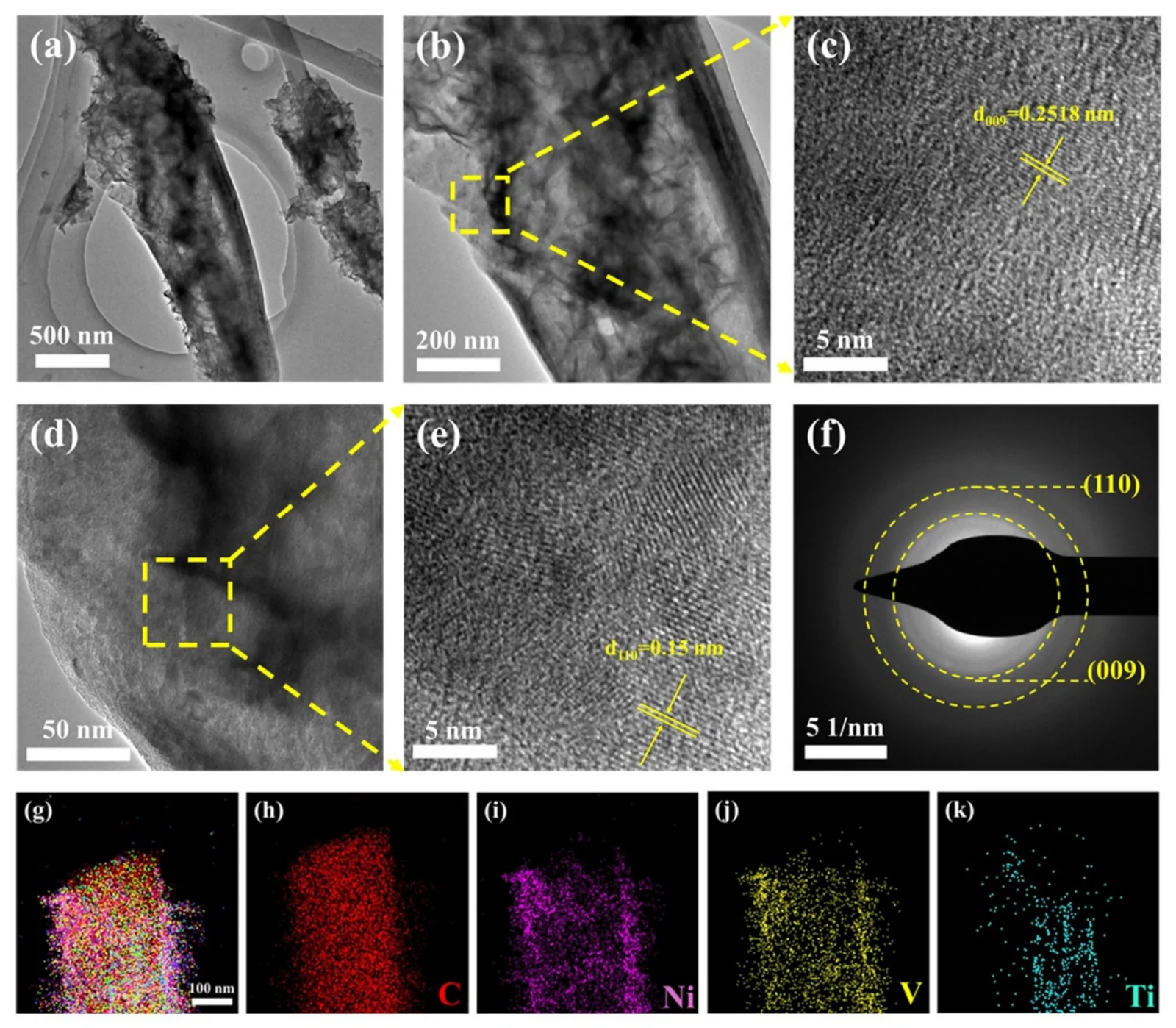
(**a**,**b**,**d**) TEM images of NiV-LDH@MXene/CNFs; (**c**,**e**) HR-TEM images of NiV-LDH@MXene/CNFs; (**f**) SAED pattern; (**g**–**k**) EDS elemental maps of NiV-LDH@MXEene/CNFs.

The microstructure of the NiV-LDH@MXene/CNFs composite is characterized by transmission electron microscopy (TEM), high-resolution transmission electron microscopy (HR-TEM), selected-area electron diffraction (SAED), and energy-dispersive X-ray spectroscopy (EDS). (Figure 3a) presents the low-magnification TEM image of the NiV-LDH@MXene/CNFs composite. Sheet-like structures uniformly distributed on the carbon fiber surfaces interweave with each other to form a network. Figure 3b,d shows that these sheet-like structures are NiV-LDH nanosheets with lateral dimensions of approximately 100–200 nm and relatively thin thickness, characteristic of layered double hydroxide. No obvious MXene sheet stacking is observed either inside or at the edges of the carbon fibers. Instead, MXene is mainly embedded in or wrapped within the carbon fiber matrix to bond with the carbon substrate. Figure 3c,e depicts the HR-TEM micrographs of a single NiV-LDH nanosheet, and the two distinct lattice fringe orientations suggest high crystallinity of the nanosheets. Using Digital Micrograph software (version 3.9, Gatan Inc., Pleasanton, CA, USA), the lattice spacings are measured to be 0.256 nm and 0.151 nm, which are assigned to the (009) and (110) planes of NiV-LDH, respectively.

The formation of NiV-LDH is corroborated by the good match with the diffraction peak positions based on XRD (X-ray diffraction). Figure 3f shows the SAED pattern of the NiV-LDH@MXene/CNFs composite. A series of distinct diffraction rings is observed, with the inner and outer rings corresponding to the (009) and (110) planes of NiV-LDH. The clear and sharp diffraction rings indicate good crystallinity of the NiV-LDH nanosheets, and the ring pattern, rather than discrete spots, suggests that the nanosheets are arranged in a polycrystalline orientation on the carbon fiber surfaces, consistent with the randomly grown morphology of the nanosheets [19]. Figure 3g–k exhibits the EDS elemental maps of the NiV-LDH@MXene/CNFs composite. Carbon (C) is mainly distributed in the fiber framework as the conductive substrate, while titanium (Ti) is uniformly distributed throughout the fiber, indicating that Ti_3_C_2_T_x_ MXene is embedded in the carbon fiber matrix. Ni and V on the fiber surfaces correspond to the NiV-LDH nanosheets, providing evidence of the growth of NiV-LDH on the carbon fibers and fabrication of the NiV-LDH@MXene/CNFs composite [20].

**Figure 4:**
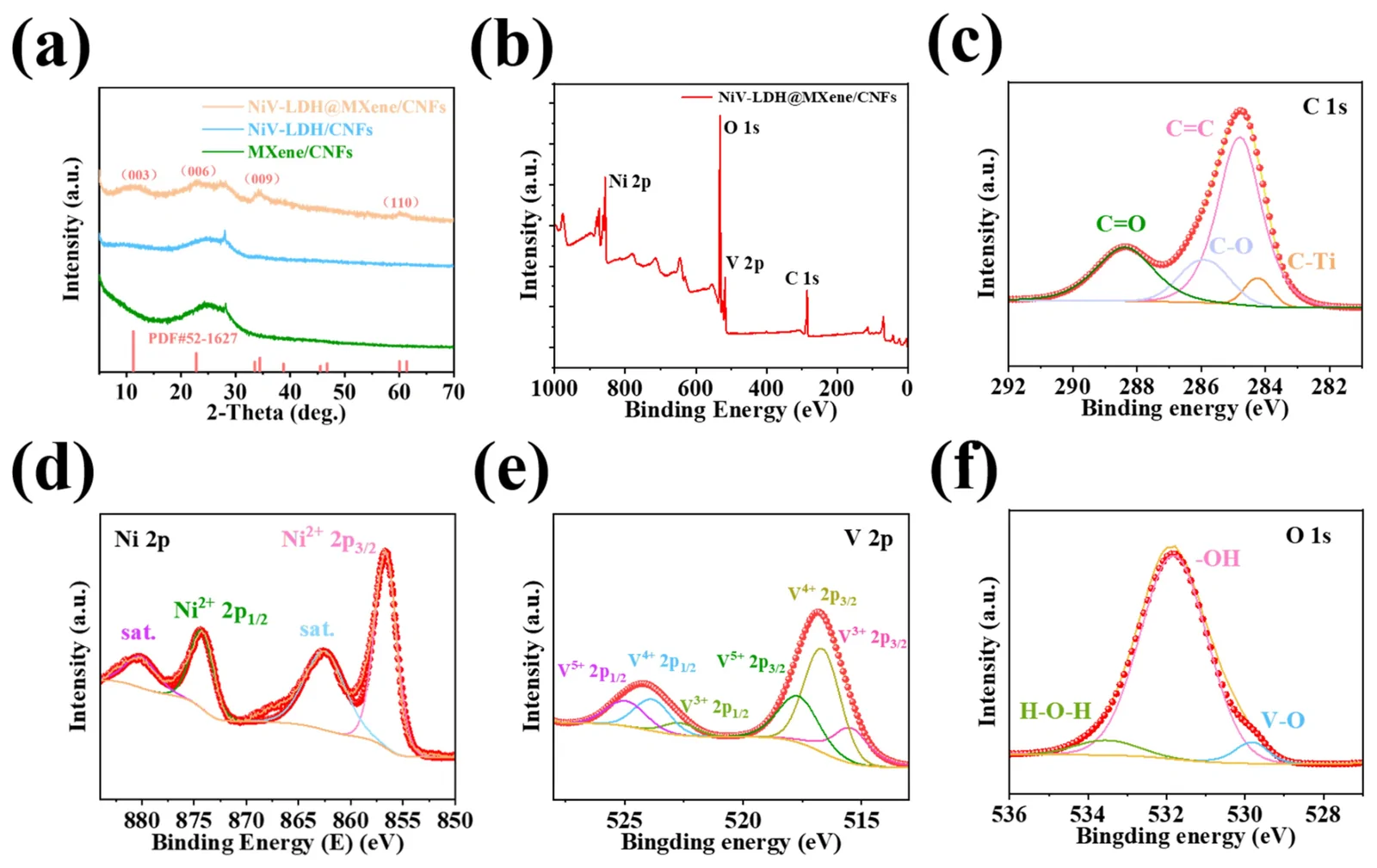
(**a**) XRD patterns of NiV-LDH@MXene/CNFs, NiV-LDH/CNFs, and MXene/CNFs; (**b**) survey spectrum and fine spectrum of NiV-LDH@MXene/CNFs; (**c**–**f**) XPS spectra of NiV-LDH@MXene/CNFs: (**c**) C 1s, (**d**) Ni 2p, (**e**) V 2p, and (**f**) O 1s.

XRD and (X-ray photoelectron spectroscopy) XPS are employed to characterize NiV-LDH@MXene/CNFs, and the results are displayed in Figure 4. Figure 4a shows the XRD patterns of NiV-LDH@MXene/CNFs, NiV-LDH/CNFs, and MXene/CNFs. All three samples display a broad peak near 24.92°, attributable to the carbon (002) facet, indicating the formation of an amorphous carbon structure by PAN carbonization [21]. For NiV-LDH/CNFs, besides the broad carbon peak, four additional diffraction peaks at 11.02°, 22.96°, 34.2°, and 60.06° can be indexed to the (003), (006), (009), and (110) planes of NiV-LDH (JCPDS card No. 52-1627), demonstrating the growth of NiV-LDH on the carbon fiber substrates [20]. The NiV-LDH@MXene/CNFs composite shows peaks from carbon, MXene, and NiV-LDH. The diffraction angles of NiV-LDH are in close agreement with those of NiV-LDH/CNFs, revealing that the NiV-LDH crystal lattice remains unaffected by MXene incorporation. Of particular note, the NiV-LDH peaks in the hybrid material are stronger than those of NiV-LDH/CNFs, likely because the ample functional groups on MXene provide extra nucleation sites for NiV-LDH growth and facilitate crystallization.

To further analyze the surface chemistry of NiV-LDH@MXene/CNFs, XPS is conducted. As shown in Figure 4b, the survey spectrum shows signals from Ni, V, O, and C. The C 1*s* core-level spectrum (Figure 4c) at 288.38, 285.86, 284.21, and 284.79 eV are assignable to C=O, C-O, C-Ti, and C-C [22]. Figure 4d shows the Ni 2*p* spectrum, in which the peaks at 856.68 eV and 874.38 eV correspond to Ni 2*p*_3/2_ and Ni 2*p*_1/2_, respectively, together with two satellite signals (marked Sat.), implying the presence of Ni^2+^ species [23]. Figure 4e displays the high-resolution V 2*p* spectrum, which has been deconvoluted into three sets of spin–orbit doublets, corresponding to V^3+^, V^4+^, and V^5+^. The peaks at 515.42 eV and 522.65 eV are assigned to V^3+^, the peaks at 516.66 eV and 523.84 eV are assigned to V^4+^, and the peaks at 517.68 eV and 524.95 eV are assigned to V^5+^, respectively. The coexistence of multiple vanadium valence states (V^3+^/V^4+^/V^5+^) is consistent with the electronic structure of NiV-LDH and is considered beneficial for the electrochemical performance. The O 1*s* spectrum (Figure 4f) can be resolved into three peaks at 529.78 eV, 531.78 eV, and 533.58 eV, which are associated with V-O, -OH, and H-O-H (hydroxyl and adsorbed oxygen species). The V-O and -OH peaks corroborate the formation of NiV-LDH hydroxide [15].

### 2.2. Electrochemical Evaluation

The electrochemical performance of the NiV-LDH@MXene/CNFs, NiV-LDH/CNFs, and MXene/CNFs electrodes was evaluated by cyclic voltammetry (CV) and galvanostatic charge–discharge (GCD) measurements in a three-electrode configuration with 6 mol L^−1^ KOH as the electrolyte at room temperature, the corresponding results are presented in Figure S1. The CV curves of MXene/CNFs, NiV-LDH/CNFs, and NiV-LDH@MXene/CNFs are displayed in Figure S1a–c at scan rates ranging from 10 to 100 mV s^−1^. For the NiV-LDH@MXene/CNFs electrode, the anodic and cathodic peaks show minor shifts toward higher and lower voltages as the scan rate rises, while maintaining distinct peak shapes, implying good reaction reversibility. Additionally, the CV loop areas steadily increase with scan rates without apparent distortion, confirming an outstanding capacitance behavior. Figure S1d–f displays the GCD responses of MXene/CNFs, NiV-LDH/CNFs, and NiV-LDH@MXene/CNFs electrodes at current densities from 1 to 10 A g^−1^. The GCD curves of both NiV-LDH/CNFs and NiV-LDH@MXene/CNFs exhibit non-linear charge/discharge plateaus typical of the Faradaic behavior and consistent with the CV redox peaks. The balanced symmetry of the GCD plots indicates good reversibility and high Coulombic efficiency [18].

**Figure 5:**
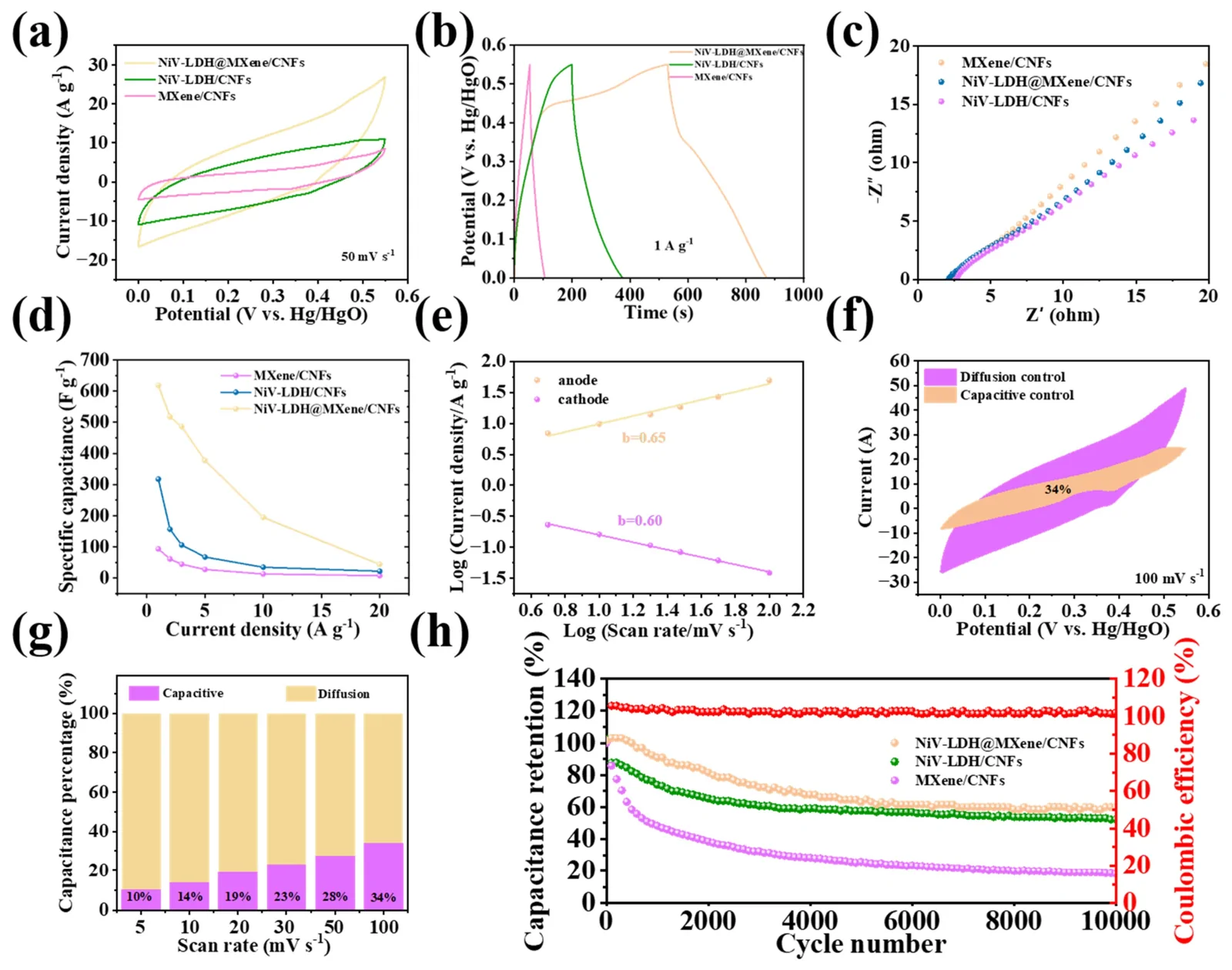
(**a**) Comparison of CV curves of NiV-LDH@MXene/CNFs, NiV-LDH@MXene/CNFs, and NiV-LDH@MXene/CNFs at a scan rate of 50 mV s^−1^; (**b**) comparison of GCD curves of NiV-LDH@MXene/CNFs, NiV-LDH@MXene/CNFs, and NiV-LDH@MXene/CNFs at a current density of 1 A g^−1^; (**c**) Nyquist plots of the materials; (**d**) specific capacitance comparison of the three electrodes at various current densities; (**e**) B-value; (**f**) capacitance contribution ratio at a scan rate of 100 mV s^−1^; (**g**) capacitance contribution ratios at different scan rates; (**h**) comparison of cycling performance at 5 A g^−1^.

Figure 5a shows the CV responses of NiV-LDH@MXene/CNFs, NiV-LDH/CNFs, and MXene/CNFs at a sweep rate of 50 mV s^−1^. Each of the three electrodes exhibits redox peaks typical of pseudocapacitive behavior. The enclosed CV areas are largest for NiV-LDH@MXene/CNFs, intermediate for NiV-LDH/CNFs, and smallest for MXene/CNFs, suggesting a capacitance ranking consistent with this order. Such a trend implies that the hybridization of NiV-LDH with the MXene/CNFs framework is beneficial to energy storage. Figure 5b shows the GCD curves of NiV-LDH@MXene/CNFs, NiV-LDH/CNFs, and MXene/CNFs at 1 A g^−1^. The two LDH-based electrodes exhibit non-linear charge/discharge plateaus in accordance with the CV redox peaks, confirming their pseudocapacitive nature. The MXene/CNFs electrode, however, exhibits a nearly triangular GCD shape characteristic of an electrical double-layer capacitor (EDLC). The discharge times are longest for NiV-LDH@MXene/CNFs, intermediate for NiV-LDH/CNFs, and shortest for MXene/CNFs, translating into the same capacitance order and proving that the MXene/CNFs scaffold improves the capacitance of NiV-LDH.

The electrode reaction kinetics is evaluated using EIS. Figure 5c and Figure S3 show the Nyquist plots of the three samples, all featuring a high-frequency semicircle and a low-frequency oblique line. The semicircle at high frequencies reflects the charge-transfer resistance (R_ct_), and the oblique line at low frequencies denotes the Warburg impedance originating from ionic transport [24]. The charge-transfer resistances of NiV-LDH@MXene/CNFs, NiV-LDH/CNFs, and MXene/CNFs are 2.224 Ω, 2.464 Ω, and 2.056 Ω, respectively. Although these values are fairly close, the MXene-modified electrode (NiV-LDH@MXene/CNFs) exhibits a markedly lower Rct and a steeper low-frequency line relative to NiV-LDH/CNFs. This shows that the MXene/CNFs scaffold affords effective electronic pathways for NiV-LDH, significantly diminishing the charge transfer impedance and enhancing the high-rate performance [25]. Figure 5d compares the specific capacitances of NiV-LDH@MXene/CNFs, NiV-LDH/CNFs, and MXene/CNFs measured at different current densities. Evidently, the NiV-LDH@MXene/CNFs electrode offers the highest capacitance among the three electrodes for all currents from 1 to 20 A g^−1^.

To further explore the charge-storage mechanism of NiV-LDH@MXene/CNFs, its CV profiles at different scan rates are analyzed based on the following equation:

$$i=av^{b}$$

where i represents the peak current, v is the scan rate, and a and b are adjustable parameters. When b approaches 1, the electrochemical process is dominated by a capacitive behavior, whereas a b value close to 0.5 indicates that the process is mainly diffusion-controlled [17]. Figure 5e displays the b values of the NiV-LDH@MXene/CNFs electrode, with the cathodic and anodic peaks yielding b values of 0.6 and 0.65, respectively, by linear fitting. These values lie between 0.5 and 1, indicating that the electrochemical process of the NiV-LDH@MXene/CNFs electrode is governed by a hybrid mechanism involving both diffusion-controlled and capacitive behaviors. To distinguish the capacitive and diffusion contributions to the total capacity, the CV data at different scan rates are analyzed by the following equation:

$$i=k_{1}v+k_{2}v^{\frac{1}{2}}$$

where i is the current at the potential v, the first term $k_{1}v$ corresponds to the capacitive-controlled process, and the second term $k_{2}v^{\frac{1}{2}}$ is related to the diffusion-controlled intercalation process. The capacitive contribution ratios were calculated at the anodic peak potential of approximately 0.46 V (vs. Hg/HgO), which was obtained from the CV curve at the lowest scan rate of 5 mV s^−1^. At this fixed potential, the current responses at different scan rates were extracted and used for the kinetic analysis. Figure 5f shows the capacitive contribution (shaded area) of the NiV-LDH@MXene/CNFs electrode at a scan rate of 100 mV s^−1^, with the calculated capacitive contribution ratio being 34%. This result is consistent with the b values, which, in the range of 0.6–0.65, indicate a hybrid control mechanism. The 34% capacitive contribution quantitatively reflects the specific proportion of capacitive behavior to the total capacity at this scan rate. Figure 5g shows the trend of the capacitive contribution ratio as a function of scan rates. From 10 to 100 mV s^−1^, the ratio shows a gradual rise from 10% to 34%. This upward trend is reasonable, as diffusion becomes more time-limited at higher scan rates, resulting in a relatively reduced diffusion contribution and a correspondingly increased proportion of surface capacitive processes [26]. Figure 5h shows the cycling stability of NiV-LDH@MXene/CNFs and NiV-LDH/CNFs at 5 A g^−1^. After 10,000 cycles, the MXene-containing electrode delivers a capacitance retention of 60%, substantially higher than the 50% of the counterpart without MXene. This outcome corroborates that the MXene/CNFs framework contributes positively to the NiV-LDH performance, particularly high-rate cycling endurance.

### 2.3. Electrochemical Properties of NiV-LDH@MXene/CNFs//AC (ASC)

**Figure 6:**
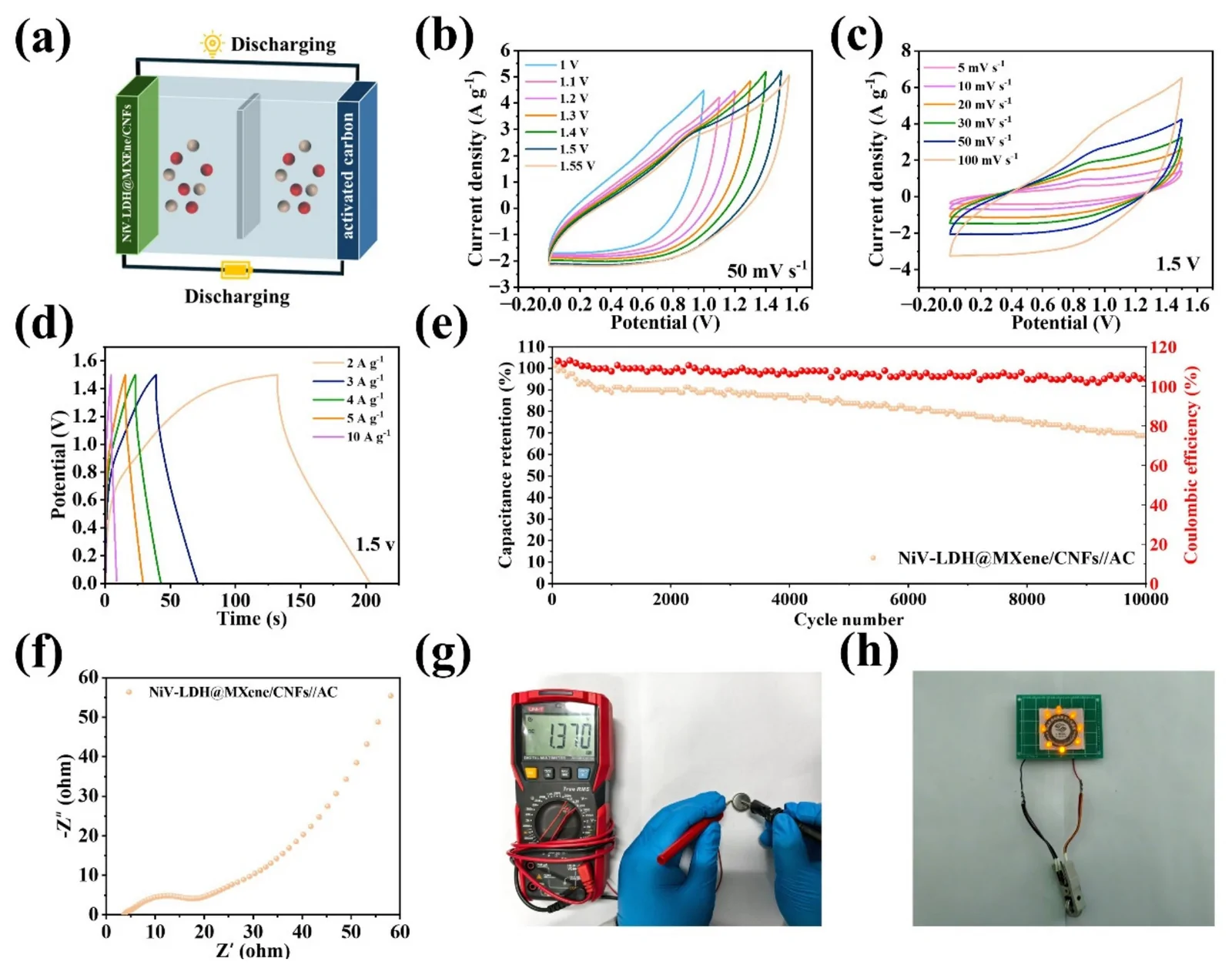
(**a**) Schematic illustration of the assembled asymmetric supercapacitor device; (**b**) CV curves of the ASC at different voltage windows at a scan rate of 50 mV s^−1^; (**c**) CV curves of the ASC at different scan rates within a voltage window of 1.5 V; (**d**) GCD curves of the ASC at different current densities; (**e**) long-term cycling performance of the ASC; (**f**) Nyquist plots of ASC; (**g**) voltage demonstration of the coin-type ASC device; (**h**) lighting test of the coin-type ASC device.

To examine the feasibility of NiV-LDH@MXene/CNFs for practical energy storage use, an asymmetric supercapacitor (ASC) cell with the NiV-LDH@MXene/CNFs//AC (activated carbon) configuration is fabricated, with NiV-LDH@MXene/CNFs as the cathode, activated carbon as the anode, cellulose film as the separator, and 6 M KOH as the electrolyte (Figure 6a). The device is subjected to thorough electrochemical testing, as shown in Figure 6. To determine the optimal operating voltage window, cyclic voltammetry is conducted at different voltage ranges. The CV curves maintain their original configuration without significant polarization when the operating voltage is raised gradually from 1.0 V to 1.5 V (Figure 6b). Upon a further voltage increase to 1.55 V, however, slight polarization becomes observable, implying that the decomposition limit of the electrolyte is being approached. On this basis, all subsequent electrochemical tests are carried out in a voltage window of 0–1.5 V. The CV curves of the asymmetric device at different scan rates in Figure 6c show a quasi-rectangular shape that is well maintained as the scan rate rises from 5 to 100 mV s^−1^, implying excellent rate capability and rapid interfacial kinetics. This is because the NiV-LDH and MXene/CNFs conductive network facilitate electron transport and ion migration [27].

GCD tests are performed to examine the capacitive response of the device. Figure 6d shows that the GCD traces at different current rates retain the favorable symmetry with no apparent charge/discharge platforms, demonstrating a storage mode consisting of pseudocapacitance and EDLC [28]. Excellent rate capability is evidenced by the discharge time results, which yield a specific capacitance of 94.67 F g^−1^ at 2 A g^−1^ (based on the total active material mass), with 28 F g^−1^ still retained at 10 A g^−1^, representing a retention of roughly 60.1%. Furthermore, the long-term cycling stability of supercapacitors is considered. Figure 6e shows the cycling longevity of the device at 5 A g^−1^. After 10,000 cycles, it preserves 68.75% of its initial capacitance, with the coulombic efficiency almost constantly at 100%, testifying to the good reversibility and stable architecture. This impressive cycling behavior is mostly due to the MXene/CNFs framework’s mechanical support and electronic conduction, consequently alleviating volume expansion and structural damage of NiV-LDH during cycling [29]. The Nyquist plot of the asymmetric cell (NiV-LDH@MXene/CNFs//AC) in Figure 6f gives an Rct of 3.473 Ω. This fairly low resistance implies that the small kinetic barrier at the electrode/electrolyte interface favors rapid electron transfer.

To further evaluate the overall energy storage performance of the NiV-LDH@MXene/CNFs//AC asymmetric supercapacitor, the Ragone plot is constructed and presented in Figure S4 [15,30,31,32]. The device delivers an energy density of 28 Wh kg^−1^ at a power density of 1401 W kg^−1^, demonstrating competitive or superior performance compared to recently reported LDH-based asymmetric supercapacitors. This outstanding energy storage capability is attributed to the synergistic effect of the conductive MXene/CNFs scaffold and the vertically aligned NiV-LDH nanosheet arrays, which facilitate rapid electron transport, shorten ion diffusion pathways, and provide abundant electrochemically active sites.

Finally, to further demonstrate the practical potential of the NiV-LDH@MXene/CNFs//AC asymmetric supercapacitor, coin-type devices are assembled and tested. As shown in Figure 6g, the open circuit voltage of a single coin-type device measured under open-circuit conditions is 1.37 V, which is close to the upper limit of the operating voltage window, indicating good charge retention capability and low leakage current of the device. Furthermore, as shown in Figure 6h, two devices connected in series provide a total voltage of 2.74 V, enough to power an LED strip with a rated voltage of about 3 V. The results directly demonstrate that the asymmetric supercapacitor has high commercial potential in the field of energy storage.

## 3. Conclusions

A flexible NiV-LDH@MXene/CNFs composite electrode with a three-dimensional network structure is prepared by in situ hydrothermal growth of NiV-LDH nanosheet arrays on electrospun Ti_3_C_2_T_x_ MXene-modified carbon nanofiber films (MXene/CNFs), which serve as the conductive substrate. This design exploits the complementary advantages of the two constituents. The MXene/CNFs scaffold ensures continuous electron transport and robust flexibility, with its abundant functional groups providing sufficient sites for NiV-LDH nucleation and growth, while the upright NiV-LDH nanosheet arrays offer plentiful active sites and facilitate rapid ion diffusion by reducing the diffusion distance. The poor intrinsic conductivity and insufficient rate capability of NiV-LDH are effectively overcome. The composite electrode shows a capacitance of 614 F g^−1^ at 1 A g^−1^ and a 60% retention over 10,000 cycles at 5 A g^−1^, confirming excellent rate and cycling capabilities. The NiV-LDH@MXene/CNFs//AC asymmetric supercapacitor, with the composite as the cathode and AC as the anode, exhibits 94.67 F g^−1^ at 2 A g^−1^, 68.75% retention after 10,000 cycles at 5 A g^−1^, and a low R_ct_ of 3.473 Ω. Two cells in series can power a 3 V LED strip, demonstrating the practical potential. The results reveal a viable design strategy for high-performance flexible supercapacitor electrodes by combining the MXene/CNFs conducting networks and LDH active phases.

## Data Availability

The data are available from the corresponding author upon reasonable request.

## Supplementary Materials

The following supporting information can be downloaded at: https://www.mdpi.com/article/10.3390/nano16161014/s1. The following supporting information can be downloaded. 1. Experimental Section. Materials, materials characterization, synthesis of Ti_3_C_2_T_x_ MXene, synthesis of MXene/CNFs, synthesis of NiV-LDH/CNFs, synthesis of NiV-LDH@MXene/CNFs, assembly of NiV-LDH@MXene/CNFs//AC asymmetric supercapacitors, electrochemical assessment 2. Supplementary figures. Figure S1. (a) CV curve of NiV-LDH@MXene/CNFs at different scan rates; (b) CV curve of NiV-LDH/CNFs at different scan rates; (c) CV curve of MXene/CNFs at different scan rates; (d) constant current charge–discharge curve of NiV-LDH@MXene/CNFs at different current densities; (e) constant current charge–discharge curve of NiV-LDH/CNFs at different current densities; (f) constant current charge–discharge curve of MXene/CNFs at different current densities. Figure S2. The flexibility demonstration of NiV-LDH@MXene/CNFs. Figure S3. The EIS data fitting and equivalent circuit diagram of NiV-LDH@MXene/CNFs. Figure S4. Ragone plot of ASC device.
