# Supplementary material for: Hierarchical NiV-LDH Nanosheet Arrays Vertically Grown on MXene-Embedded Carbon Nanofibers for High-Performance Flexible Supercapacitors
Source: Nanomaterials (Basel). 2026 Aug 17;16(16):1014. doi: 10.3390/nano16161014 (PMC13516632; doi:10.3390/nano16161014)
Supplement: Supplementary file 1 [file nanomaterials-16-01014-s001.zip › nanomaterials-4471872-supplementary.pdf]

Hierarchical NiV-LDH Nanosheet Arrays Vertically Grown on MXene-Embedded Carbon Nanofibers for High-Performance Flexible Supercapacitors

*Deyang Zhang, * [a] Wenbo Guo, [a] Binhe Feng, [a] Yikai Ge, [a] Jinbing Cheng [b] and Paul K. Chu [c]*

[a.] *Henan Joint International Research Laboratory of New Energy Storage Technology, Xinyang Normal University, Xinyang 464000, PR China*

[b.] *Henan International Joint Laboratory of MXene Materials Microstructure, Collaborative Innovation Center of Intelligent Explosion-proof Equipment of Henan Province, Nanyang Normal University, Nanyang 473061, PR China*

[c.] *Department of Physics, Department of Materials Science & Engineering, and Department of Biomedical Engineering, City University of Hong Kong, Tat Chee Avenue, Kowloon, Hong Kong, China*

*Corresponding author: zdy@xynu.edu.cn

Experimental Section

Materials

The Ti_3AlC_2 powder (400 mesh) was purchased from Enwang New Materials Technology Co., Ltd. The chemical reagents used in this work, including hydrofluoric acid (49%), hydrochloric acid (12 M), lithium fluoride, N,N-dimethylformamide (DMF, 98%), polyacrylonitrile (PAN, Mw = 150,000), nickel chloride ($NiCl_2$), citric acid ($C_6H_8O_7$), urea ($CO(NH_2)_2$), and potassium hydroxide (KOH), were all supplied by Sinopharm Chemical Reagent Co., Ltd. Vanadium trichloride (VCl_3, 96%) was purchased from Aladdin Biochemical Technology Co., Ltd. All reagents were of analytical grade and used as received without further purification.

Materials characterization

The crystalline structure and phase of the composites were identified by X-ray

diffraction (XRD, Bruker D2 PHASER) using Cu-Kα (λ = 1.5418 Å) radiation at 40 kV, 40 mA, and 2θ between 5° and 80° at room temperature. The chemical composition was determined by X-ray photoelectron spectroscopy (XPS, K-ALPHA 0.5 eV) with a resolution of 0.3-0.5 eV using a monochromatic aluminum X-ray source. The morphology was examined by field-emission scanning electron microscopy (SEM, Hitachi S-4800) and transmission electron microscopy (TEM, FEI Tecnai G2 F20), and elemental analysis was carried out by energy-dispersive X-ray spectroscopy (EDS, Bruker QUANTAX) on the TEM.

Synthesis of $Ti_3C_2T_x$ MXene

$Ti_3C_2T_x$ nanosheets were synthesized via selective etching of Ti_3AlC_2 using a LiF/HCl mixture, followed by exfoliation. In a typical procedure, LiF (1 g) was dissolved in 30 mL of 9 mol/L HCl and heated to 36 °C under stirring. Once the solution became clear, 5 mL of 40% HF was introduced. Subsequently, 1 g of Ti_3AlC_2 MAX powder was added and the reaction was allowed to proceed for 12 h. The resulting black suspension was repeatedly washed with deionized water and centrifuged at 5,000 rpm until the supernatant reached a pH value of ⩾ 6. The collected dark green mud-like sediment was then transferred to a sealed bottle, placed under an argon atmosphere, and ultrasonicated for 1 h. After further washing with deionized water and centrifugation at 10,000 rpm, the product was collected. To prepare the electrospinning precursor, the obtained product was washed three times with DMF and the final concentration was adjusted to 20 mg mL^{-1}.

Synthesis of MXene/CNFs

MXene/CNFs were fabricated via electrospinning. Specifically, 0.6 g of PAN powder was added to 5 mL of the $Ti_3C_2T_x$ MXene solution and stirred overnight to form a homogeneous spinning precursor. The spinning solution was then loaded into a 5 mL plastic syringe equipped with an 18-gauge flat-tip needle. The electrospinning parameters were set as follows: applied voltage of 20 kV, tip-to-collector distance of 12 cm, feed rate of 0.4 mL h^{-1}, and ambient humidity maintained below 30%. The as-collected nanofiber films were subsequently placed in a muffle furnace and pre-oxidized at 250 °C for 2 h with a ramping rate of 2 °C min^{-1}, followed by carbonization at 800 °C for 2 h under argon atmosphere with the same ramping rate of 2 °C min^{-1}.

Synthesis of NiV-LDH/CNFs

PAN/CNFs carbon nanofibers were first prepared as follows. A total of 0.6 g of PAN powder was dispersed in 5 mL of DMF and stirred overnight to obtain a homogeneous solution, which was then subjected to electrospinning. The spinning solution was loaded into a 5 mL plastic syringe equipped with an 18-gauge flat-tip needle. The electrospinning parameters were set as follows: applied voltage of 20 kV, tip-to-collector distance of 12 cm, feed rate of 0.4 mL h^{-1}, and ambient humidity maintained below 30%. The as-collected nanofiber films were subsequently pre-oxidized in a muffle furnace at 250 °C for 2 h with a ramping rate of 2 °C min^{-1}, followed by carbonization at 800 °C for 2 h under argon atmosphere with the same ramping rate of 2 °C min^{-1}. The resulting films were then cut into rectangular pieces of 1 cm × 1.5 cm for later use. Subsequently, 1 g of nickel chloride hexahydrate

($NiCl_2·6H_2O$), 3 g of urea, 0.03 g of citric acid monohydrate ($C_6H_8O_7·H_2O$), and 0.3 g of vanadium trichloride (VCl_3) were dispersed in 50 mL of deionized water and stirred vigorously for 30 min. The resulting solution was transferred into a Teflon-lined stainless steel autoclave, into which the pre-cut carbon fiber pieces were also placed. The hydrothermal reaction was conducted at 120 °C for 10 h. After the reaction, the carbon fiber pieces were taken out, carefully rinsed, and immersed in deionized water for 30 min to thoroughly remove any residual surface contaminants. Finally, the samples were dried in a vacuum oven at 60 °C for 1 h.

Synthesis of NiV-LDH@MXene/CNFs

The preparation procedure for NiV-LDH@MXene/CNFs was identical to that described in the previous subsection for NiV-LDH/CNFs, except that the conventional PAN/CNFs carbon fibers were replaced by MXene/CNFs as the substrate.

Assembly of NiV-LDH@MXene/CNFs//AC asymmetric supercapacitor

Activated carbon was employed as the negative electrode active material, with nickel foam as the current collector, carbon black as the conductive additive, and polytetrafluoroethylene (PTFE) as the binder. The composite negative electrode was fabricated via a blending–coating process for use in the asymmetric supercapacitor. The mass of the positive electrode active material was determined based on the mass difference between the NiV-LDH@MXene/CNFs composite electrode and the pristine MXene/CNFs substrate. To satisfy the charge balance principle during device assembly, the activated carbon negative electrode was precisely weighed, and the

mass ratio of the positive to negative electrodes was calculated according to the relevant formula, ensuring equal charge storage capacity between the two electrodes. Finally, the asymmetric coin-type supercapacitor was assembled using the aforementioned activated carbon negative electrode and the NiV-LDH@MXene/CNFs positive electrode, with cellulose membrane as the separator and 6 mol L^{-1} KOH as the electrolyte.

Electrochemical assessment

The electrochemical performance of MXene/CNFs, NiV-LDH/CNFs, and NiV-LDH@MXene/CNFs was evaluated using a conventional three-electrode system in 6 M KOH electrolyte at 25 °C. All measurements were conducted on a CHI660E electrochemical workstation, with a platinum plate and a Hg/HgO electrode serving as the counter and reference electrodes, respectively.

Cyclic voltammetry (CV) was performed in the voltage ranges between -1 and 0 as well as 0 and 1 V. The GCD curves were acquired according to the CV window. Electrochemical impedance spectroscopy (EIS) was conducted at frequencies between 0.01 Hz and 100 kHz. The specific capacitance (Cm, F g^{-1}) was calculated by the following equation:

$$C_m = \frac{I \times \Delta t}{m \times \Delta V}$$

where I (A) is the applied current, Δt (s) is the discharging time, ΔV (V) is the discharging potential range, and m (g) is the total mass of active materials. The theoretical pseudo-capacitance was calculated as follows:

$$C = \frac{n \times F}{M \times V}$$

where n (mol) is the number of electrons transferred in the redox reaction, M (g mol^{-1}) is the molar mass of the materials, F (C. mol^{-1}) is Faraday's constant, and V (V) is the operating voltage window, respectively. The charge balance between the two electrodes should follow the relationship $q^+ = q^-$, where q is the charge stored in the electrode calculated by the following equation:

$$q = C_m \times \Delta E \times m$$

where Cm (F g^{-1}) is the specific capacitance, ΔE (V) is the potential range in the charging/discharging process, and m (g) is the mass loading of the active materials. The ideal mass ratio of the active materials on the positive (NiV-LDH@MXene/CNFs) and the negative electrode (AC) in the asymmetric supercapacitor (m^+/m^-) was calculated by the following equation:

$$\frac{m^+}{m^-} = \frac{\Delta E_- \times C_-}{\Delta E_+ \times C_+}$$

The energy density (E) and power density (P) were calculated by the following equations:

$$E = \frac{1}{2\times 3.6} C_m (\Delta V)^2 \text{ and}$$

$$P = \frac{E \times 3600}{\Delta t}$$

where E (Wh kg^{-1}) is the energy density, Cm (F g^{-1}) is the specific capacitance, ΔV (V) is the operating potential window, P (kW kg^{-1}) is the power density, and Δt(s) is the discharging time.

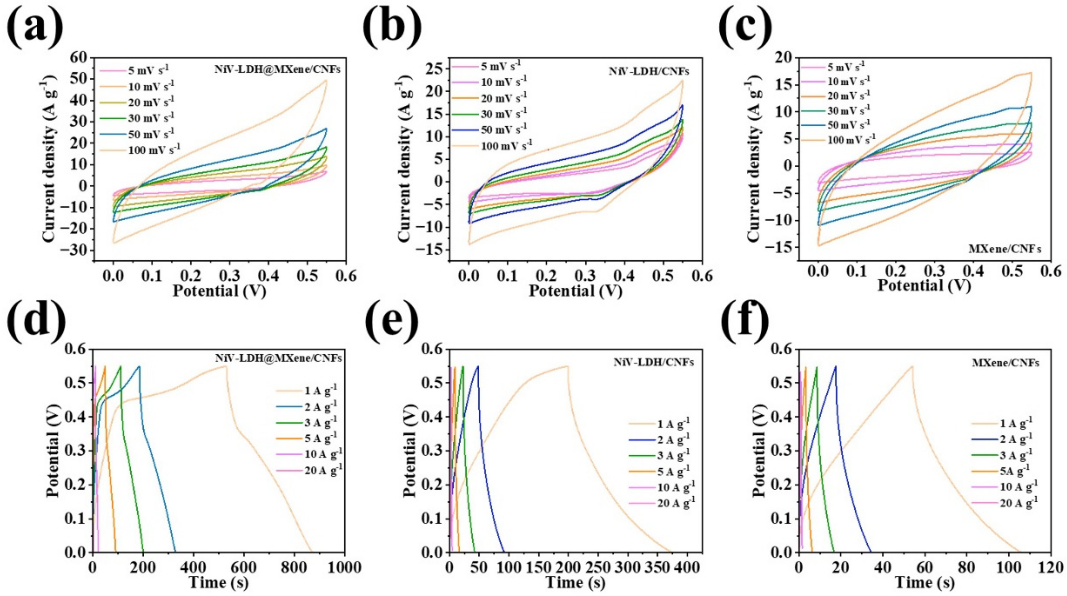

Figure S1. (a) CV curve of NiV-LDH@MXene/CNFs at different scan rates; (b) CV curve of NiV-LDH/CNFs at different scan rates; (c) CV curve of MXene/CNFs at different scan rates; (d) constant current charge–discharge curve of NiV-LDH@MXene/CNFs at different current densities; (e) constant current charge–discharge curve of NiV-LDH/CNFs at different current densities; (f) constant current charge–discharge curve of MXene/CNFs at different current densities.

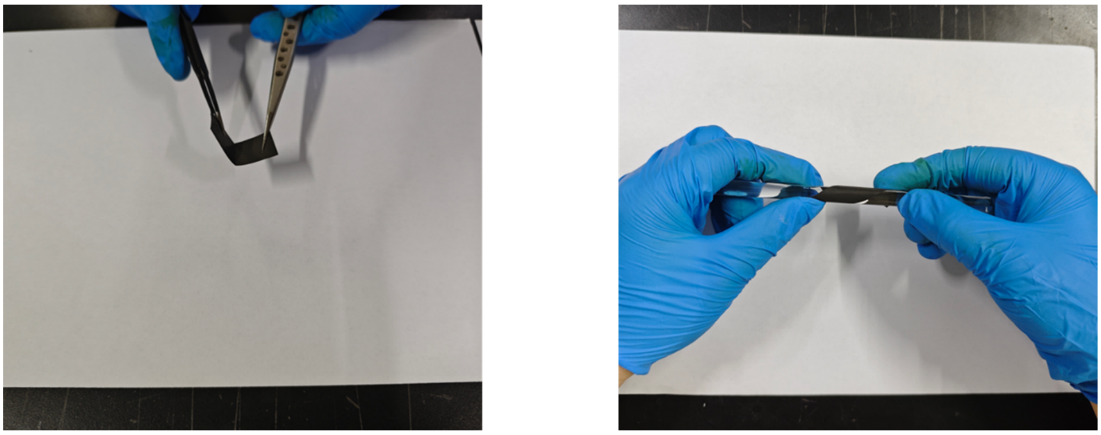

Figure S2. The flexibility demonstration of NiV-LDH@MXene/CNFs.

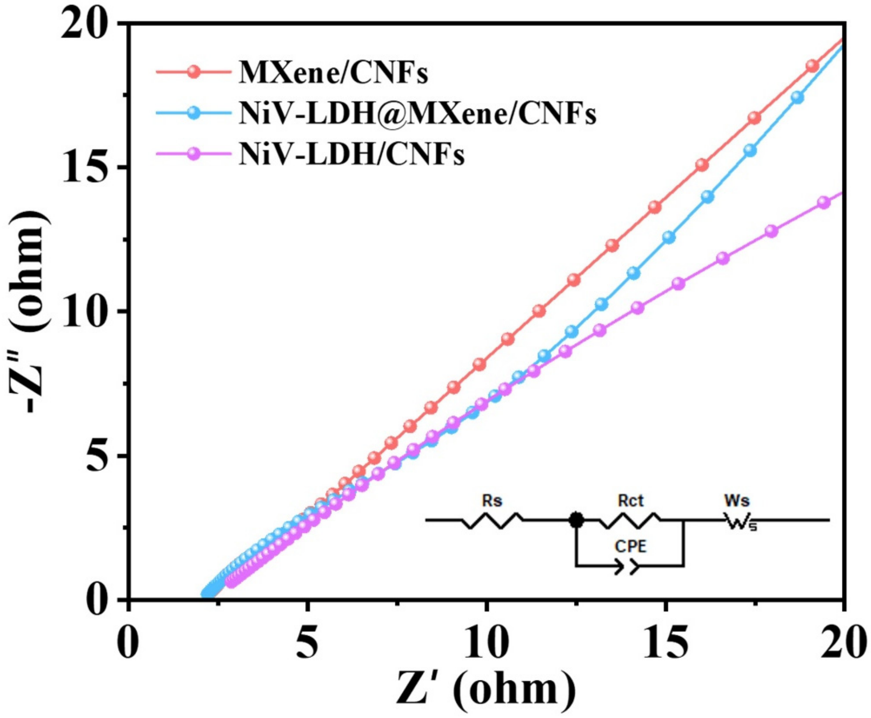

Figure S3. The EIS data fitting and equivalent circuit diagram of NiV-LDH@MXene/CNFs.

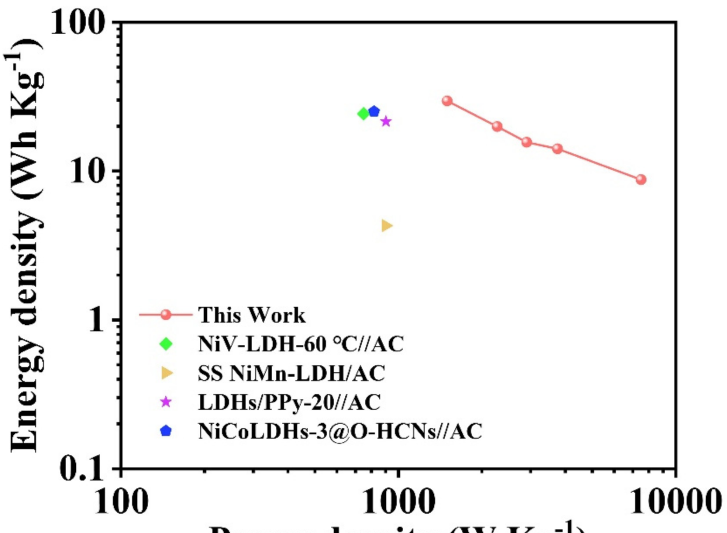

Figure S4. Ragone plot of ASC device.
